# Fast Positioning Model and Systematic Error Calibration of Chang’E-3 Obstacle Avoidance Lidar for Soft Landing

**DOI:** 10.3390/s22197366

**Published:** 2022-09-28

**Authors:** Donghong Wang, Xingfeng Chen, Jun Liu, Zongqi Liu, Fengjie Zheng, Limin Zhao, Jiaguo Li, Xiaofei Mi

**Affiliations:** 1 Beijing Institute of Tracking and Telecommunications Technology, Beijing 100094, China; 2 Aerospace Information Research Institute, Chinese Academy of Sciences, Beijing 100094, China; 3 School of Space Information, Space Engineering University, Beijing 101416, China; 4 School of Surveying and Land Information Engineering, Henan Polytechnic University, Jiaozuo 454000, China

**Keywords:** Chang’E-3, LiDAR, intrinsic parameters, geometric calibration, planar target plate

## Abstract

Chang’E-3 is China’s first soft landing mission on an extraterrestrial celestial body. The laser Three-Dimensional Imaging (TDI) sensor is one of the key payloads of the Chang’E-3 lander. Its main task is to provide accurate 3D lunar surface information of the target landing area in real time for the selection of safe landing sites. Here, a simplified positioning model was constructed, to meet the accuracy and processing timeline requirements of the TDI sensor of Chang’E-3. By analyzing the influence of TDI intrinsic parameters, a permanent outdoor calibration field based on flat plates was specially designed and constructed, and a robust solution of the geometric calibration adjustment was realized by introducing virtual observation equations for unknowns. The geometric calibration and its absolute and relative positioning accuracy verification were carried out using multi-measurement and multi-angle imaging data. The results show that the error of TDI intrinsic parameters will produce a false obstacle with a maximum height of about 1.4 m on the plane, which will cause the obstacle avoidance system of Chang’E-3 to fail to find a suitable landing area or find a false flat area. Furthermore, the intrinsic parameters of the TDI have good stability and the accuracy of the reconstructed three-dimensional surface can reach about 4 cm after error calibration, which provides a reliable terrain guarantee for the autonomous obstacle avoidance of the Chang’E-3 lander.

## 1. Introduction

Chang’E-3 (CE-3), successfully launched on 2 December 2013, was China’s first soft landing mission on an extraterrestrial celestial body. After five days of flight, CE-3 entered lunar orbit, and finally reached the closest point of 15 km to the moon on December 14; then, it started the most critical and exciting lunar soft landing process. The process included seven successive stages: landing preparation, primary deceleration, quick adjusting, approaching, hovering, hazard avoidance, and constant low-velocity descent [1].

The coarse-to-fine relay obstacle avoidance system unique to the CE-3 lander started to work from the fourth stage, that is, the approaching stage. It used the image captured by a spaceborne optical camera to detect large obstacles (rocks or potholes with a diameter greater than 1 m), to find the target landing area, and then to descend to a height of about 100 m and hover. During the hovering phase, the CE-3 lander used the laser Three-Dimensional Imaging (TDI) sensor to collect accurate 3D terrain data of the target landing area, and carried out detection of small obstacles (rocks or potholes with a height of more than 20 cm) to determine a safe landing site, and then trigger the next precise obstacle avoidance process [1]. The main task of TDI was to scan the target landing area of 50×50 m^2^ below at a height of about 100 m from the lunar surface, and complete the three-dimensional reconstruction of the lunar surface information of the landing area within 0.25 s.

The TDI sensor is a dual galvanometer-based multi-channel Light Detection And Ranging (LiDAR) sensor which can achieve a total field of view of about 30° × 30°. Xu et al. briefly discussed the working principle, positioning model, and accuracy of dual galvanometer-based multi-channel imaging LiDAR. On this basis, he proposed a correction method for the systematic distortion [2]. However, due to the limitation of computing ability and processing time of the CE-3 soft landing system, the strict positioning equations given in Ref. [2] are too complex to meet the requirements of on-board real-time processing. Therefore, it is necessary to simplify the strict positioning equations and carry out geometric calibration for the system parameters in simplified model.

Early studies on LiDAR geometric calibration have used photogrammetric methods for reference. Point-like objects as control and tie elements to construct error equations have been used, and least squares adjustment has been carried out to solve them [3]. However, accurate determination of the laser footprints of control and tie points is difficult to achieve due to the discrete sampling characteristics of LiDAR and the limitations of the laser footprint density. Most of these methods have used approximations for identifying control or tie points. For example, the original point cloud is interpolated as a raster image, and then control or tie points are extracted from this height image. García-Gómez presented a novel calibration method for solid-state LiDAR devices based on a geometrical description of their scanning system, which had a variable angular resolution. The process effectively improved the accuracy of the existing scanning system [4].

Some scholars have designed special-shaped targets for LiDAR system calibration [5,6,7,8]. Filin proposed a method of LiDAR geometric calibration using flat features, and established a mathematical model based on the coplanar constraints of laser footprints. It has been widely used to calibrate intrinsic and mounting parameters of static, vehicle-borned, and airborne LiDAR [9,10,11,12]. Heinz et al. investigated the design of a planar-based permanent calibration field and used I for the geometric calibration of vehicle-borned LiDAR [13]. Another focus of LiDAR geometric calibration research is to determine the error models that describes systematic errors, which can be divided into physical and empirical models. Physical models model the errors of LiDAR based on physical parameters, such as ranging, angle measurement, and quadrature errors, while empirical models are based on empirical parameters [14,15,16,17]. To reduce the correlation among model parameters, these parameters can be treated as observations with prior information [18,19,20], or the strongly correlated parameters are grouped and calibrated step by step [21,22]. 

In summary, although there are already mature methods for LiDAR geometric calibration that can be used, the CE-3 laser 3D imaging sensor, studied in this paper, has a significantly different imaging principle from conventional LiDAR, which results in more complex positioning equations and geometric calibration models, and also needs to balance complexity and computational efficiency to meet the requirements of the CE-3 relay obstacle avoidance system. Therefore, in this paper, first, the strict positioning model of the CE-3’s TDI is simplified, then a permanent calibration field is designed and built based on its system parameter characteristics, and finally geometric calibration and accuracy validation are carried out by using multi-measurement and multi-angle scan data.This research provides a systematic solution for the geometric calibration of multi-channel LiDAR, and provides a valuable obstacle avoidance scheme for the soft landing of other sensors on extraterrestrial celestial bodies.

## 2. Positioning Model of CE-3 TDI and Its Simplification

**Figure 1:**
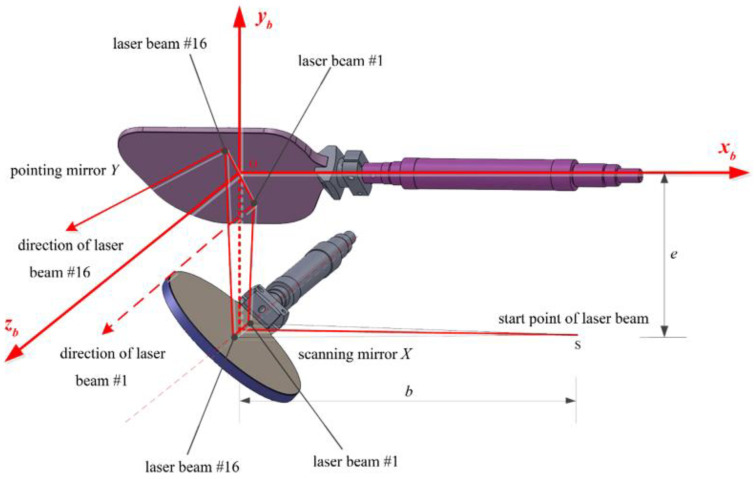
TDI dual galvanometer scanning principle. From point S, 16 laser beams are instantaneously emitted to the scanning mirror X (the 16 laser beams are coplanar), they are reflected by the scanning mirror X and then reach the pointing mirror Y, then fly to the target area after the second reflection. The scanning mirror X and the pointing mirror Y are perpendicular to each other. $o-x_{b}y_{b}z_{b}$ is the TDI body coordinate system, *b* is the distance from the laser emission point to the scanning mirror X, and *e* is the distance from the scanning mirror X center to the pointing mirror Y center.

The CE-3’s TDI is a dual galvanometer based on multi-channel LiDAR. The pulsed laser emitted by the laser is divided into 16 beams through the grating, which is received by the multi-array detector in parallel, and the timing circuit obtains the ranging information. The front end of the optical path uses dual galvanometers (called scanning mirrors and pointing mirrors) to realize two-dimensional field of view scanning [2,3]. The two scanning mirrors can realize 30° × 30° full field of view scanning in 0.1 s, as shown in Figure 1.

The derivation of the TDI laser footprint positioning equation is complex and will not be developed specifically here; its rigorous analytical formula is given in Ref. [2]. The TDI body coordinate system $o-x_{b}y_{b}z_{b}$ is defined as follows: the origin O is located at the centre of the pointing mirror Y, the $x_{b}$ axis is in the opposite direction (left) along the outgoing light of the laser, the $y_{b}$ axis passes through the center of the scanning mirror X to the center of the pointing mirror Y (up), and the $z_{b}$ axis, $x_{b}$ and $y_{b},$ form the right hand coordinate system (forward). In this coordinate system, the coordinates of the kth(k=1,2,⋯,16) laser beam’s footprint ($x_{k},y_{k}$,$z_{k}$) are calculated as [2]:

(1) $$\left[\begin{array}{c} x_{k}\\ y_{k}\\ z_{k} \end{array}\right]=\left[\begin{array}{c} x_{sk}\\ y_{sk}\\ z_{sk} \end{array}\right]-L_{k}cos\theta_{k}\left[\begin{array}{c} sin2\theta_{x}\\ cos2\theta_{x}sin2\theta_{y}+cos2\theta_{y}tan\theta_{k}\\ -cos2\theta_{x}cos2\theta_{y}+sin2\theta_{y}tan\theta_{k} \end{array}\right]$$

among them,

$$L_{k}=\rho_{k}-\frac{b}{cos\theta_{k}}-\sqrt{{\left(x_{sk}-0\right)}^{2}+{\left(y_{sk}+e\right)}^{2}+{\left(z_{sk}-btan\theta_{k}\right)}^{2}}$$

$$\{\begin{array}{c} x_{sk}=\frac{-sin2\theta_{x}\left(btan\theta_{k}+etan\left(\frac{\pi }{4}+\theta_{y}\right)\right)}{cos2\theta_{x}tan\left(\frac{\pi }{4}+\theta_{y}\right)-tan\theta_{k}}\\ y_{sk}=\frac{tan\theta_{k}\left(e+bcos2\theta_{x}\right)}{cos2\theta_{x}tan\left(\frac{\pi }{4}+\theta_{y}\right)-tan\theta_{k}}\\ z_{sk}=\frac{tan\theta_{k}tan\left(\frac{\pi }{4}+\theta_{y}\right)\left(e+bcos2\theta_{x}\right)}{cos2\theta_{x}tan\left(\frac{\pi }{4}+\theta_{y}\right)-tan\theta_{k}} \end{array}$$

($x_{sk},y_{sk}$, $z_{sk}$) are the position of the kth laser beam’s footprint on mirror Y. $\rho_{k}$ is the flight distance of the kth laser beam, namely the range observation, $L_{k}$ is the distance from body frame origin O to ground laser footprint, b,e are known constants for the TDI device. $\theta_{x}$ and $\theta_{y}$ are the swing angles of the scanning mirrors on $x_{b}$ and $y_{b}$ axes, respectively, which are obtained by converting the voltage output values of the scanning motors corresponding to the two scanning mirrors, and their values range from −15° to +15°. $\theta_{k}$ is the angle between the kth element laser beam and the $x_{b}$ axis, when k = 1, 2, …, 15, 16, the sequence is −15, −13, …, 13, 15 mrad. 

As can be seen, the strict positioning model shown in Equation (1) is very complex, involving many complex trigonometric operations. Due to the strict time constraint of the CE-3 soft landing system for the whole process of lunar surface terrain data acquisition and processing, it cannot be directly burned into the microcontroller for operation. The basic principle of model simplification is to replace the complex trigonometric equation with a Taylor expansion of finite order based on the range of values of the independent variables, and to ensure that the errors brought by the expression simplification are acceptable in specific application scenarios. For this purpose, simulation data were generated under the conditions of vertical imaging at 100 m above flat ground using TDI design parameters. Based on the simulated data, the order of the Taylor series can be determined by judging the errors introduced by truncating the Taylor polynomials.

The specific method is: As the value of $\theta_{k}$ is small, the theoretical value is (−15~15 mrad), $\theta_{k}$ can be used to replace $tan\theta_{k}$. As the imaging distance is around 100 m, the Taylor expansion is used to replace the sine and cosine values of $\theta_{x}$ and $\theta_{y}$. Taking $\theta_{x}$ as an example: $sin\theta_{x}=\theta_{x}-\frac{1}{6}{\theta_{x}}^{3}$ and $cos\theta_{x}=1-\frac{1}{2}{\theta_{x}}^{2}+\frac{1}{24}{\theta_{x}}^{4}$, simplifying the $L_{k}$ expression to $L_{k}=\rho_{k}-b-e$. 

Under the above assumptions, the strict positioning equations of the TDI are simplified to obtain a simplified version for fast positioning computation. In the simplified model, the coordinates of the laser footprint in the TDI body coordinate system $x_{b}={\left(x_{k},y_{k},z_{k}\right)}^{T}$ can be expressed as:

(2) $$\left[\begin{array}{c} x_{k}\\ y_{k}\\ z_{k} \end{array}\right]=\left[\begin{array}{c} x_{sk}\\ y_{sk}\\ z_{sk} \end{array}\right]-L_{k}\left(1-\frac{\theta_{k}{}^{2}}{2}\right)\left[\begin{array}{c} n_{x}\\ n_{y}\\ n_{z} \end{array}\right]$$

among them,

$$x_{sk}=\frac{e\left(6\theta_{x}-4\theta_{x}{}^{3}\right)}{3-6\theta_{x}{}^{2}+2\theta_{x}{}^{4}},\;y_{sk}=z_{sk}=0,\;L_{k}=\rho_{k}-b-e$$

$$\left[\begin{array}{c} n_{x}\\ n_{y}\\ n_{z} \end{array}\right]=\left[\begin{array}{c} -\left(2\theta_{x}-4\theta_{x}{}^{3}/3\right)\\ \left(1-2\theta_{x}{}^{2}+2\theta_{x}{}^{4}/3\right)\left(2\theta_{y}-4\theta_{y}{}^{3}/3\right)+\left(1-2\theta_{y}{}^{2}+2\theta_{y}{}^{4}/3\right)\theta_{k}\\ -\left(1-2\theta_{x}{}^{2}+2\theta_{x}{}^{4}/3\right)\left(1-2\theta_{y}{}^{2}+2\theta_{y}{}^{4}/3\right)+\left(2\theta_{y}-4\theta_{y}{}^{3}/3\right)\theta_{k} \end{array}\right]$$

Based on the simulation data, the maximum deviation introduced on plane coordinates is about 1.6 cm and the maximum deviation caused in the depth direction is about 0.6 cm by comparing the coordinates computed by the strict model and the simplified one. Considering the main task of TDI, the error in the depth direction is the greatest concerned. However, as compared with the main task objective of detecting obstacles with a relative height difference greater than 20 cm, the depth error of 0.6 cm caused by the simplification of the TDI positioning model is acceptable [1].

## 3. TDI Intrinsic Parameter Calibration Based on Plane Targets

From Equation (2) combined with the design principle and application scenario of TDI, it can be seen that the systematic error sources affecting the internal accuracy of the TDI point cloud are mainly the distance observation $L_{k}$, the scan angles $\theta_{x}$ and $\theta_{y}$, and the probe emission angle $\theta_{k}$. Because of the strong correlation between $\theta_{k}$ and $\theta_{x}$, their effect on the geographical location can be substituted for each other, and one of them can be chosen. Therefore, the actual intrinsic parameters involved in the calibration of the TDI are $L_{k}\left(k=1,2,\cdots ,16\right)$, scan angle $\theta_{x}$ and $\theta_{y}$, a total of 18 parameters. Only constant error corrections are made for each intrinsic parameter.

### 3.1. TDI Geometric Calibration Model Based on Planar Target Plate

In this paper, some parametric geometric surfaces with known positioning parameters are used as the control element for the geometric calibration of the TDI intrinsic parameters, specifically planar control target plates. The planar target plates used for calibration should be placed in front of the TDI, and their distribution preferably covers the entire field of view of the TDI evenly. The analytical equation of the planar target plate can be expressed as:

(3) f(x)=AX+BY+CZ+D=0

Among them, *A*, *B*, *C*, and *D* are the geometric parameters of the plane target plate, which can be calculated in advance by the coordinates of some reflective marker points laid out on the planar target, and then treated as known values in the subsequent geometric calibration process, and $x={\left(X,Y,Z\right)}^{T}$ is the ground coordinate. The calculation method of the *A*, *B*, *C*, and *D* parameters is as follows: Firstly, the ground coordinates of each reflective marker point are measured using a total station with millimeter-level accuracy, then they are substituted into Equation (3), and finally the least squares adjustment method is adopted to solve them. The relationship between the TDI body coordinate system and the ground coordinate system can be expressed as translation and rotation in space, hereinafter, it is called the datum transformation:

(4) $$x=x_{0}+R_{mis}\left(\omega ,\varphi ,\gamma \right)\cdot x_{b}$$

where (ω,φ,k) are the Euler angles from the ground coordinate system to the TDI body coordinate system, and $x_{0}={\left(X_{0},Y_{0},Z_{0}\right)}^{T}$ is the ground coordinate of the origin of the TDI body coordinate system. Let:

$$\bar{x}=R_{mis}\left(\omega ,\varphi ,\gamma \right)\cdot x_{b}={\left(\bar{X},\bar{Y},\bar{Z}\right)}^{T}$$

Substituting Equation (4) into Equation (3), the observation equation of geometric calibration of intrinsic parameters in TDI based on planar control is as follows:

(5) $$A\left(X_{0}+\bar{X}\right)+B\left(Y_{0}+\bar{Y}\right)+C\left(Z_{0}+\bar{Z}\right)+D=0$$

When applying Equation (5) for system calibration, there are two types of unknowns included: the TDI intrinsic parameters and the datum transformation parameters $\left(X_{0},Y_{0},Z_{0},\omega ,\varphi ,\gamma \right)$ between the TDI body coordinate system and the ground coordinate system.

After linearization of Equation (5), the error equation for parameter calibration of TDI can be obtained:

(6) $$v=a_{1}\Delta \theta_{x}+a_{2}\Delta \theta_{y}+a_{3}\Delta L_{k}+a_{4}\Delta X_{0}+a_{5}\Delta Y_{0}+a_{6}\Delta Z_{0}+a_{7}\Delta \omega +a_{8}\Delta \varphi +a_{9}\Delta \gamma -l$$

where the subscript k(k=1,2,⋯,16) indicates the channel number. For each laser footprint on a flat target plate, one error equation can be constructed according to Equation (6).

### 3.2. Determination of Initial Values and Treatment of Correlations

**Figure 2:**
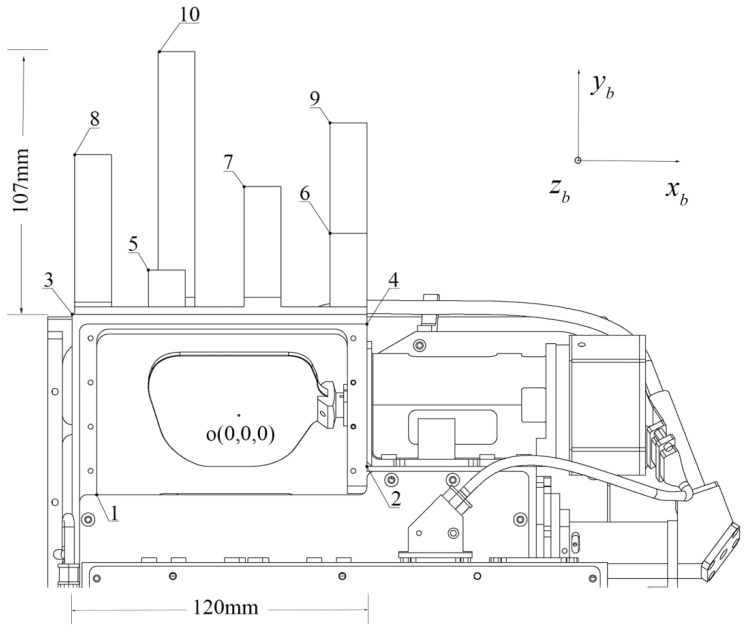
Additional structural part and calibration points mounted on the top of the TDI system. Points 1 to 10 are specially set marker points for determining the datum transformation parameters between the TDI body coordinate system and the ground coordinate system. Among them, points 1 and 2 are located on the TDI device itself, and points 3 to 10 are located on the structural part installed at the top of the TDI. The structure part itself is not part of the TDI, it is only used to determine the rigid transformation parameters. The height difference of the structural parts is 107 mm, and the width of the TDI laser exit window is about 120 mm. Point “o” is the origin of the TDI body coordinate system, and $x_{b}$,$\;y_{b}$ and $z_{b}$ are the coordinate axes.

The two types of unknowns in Equation (6) are strongly correlated, for example, $\theta_{y}$ and ω, and the least squares adjustment tends to converge to a local optimum solution. In order to solve this problem, the initial values of the datum transformation parameters need to be determined with high accuracy before the calibration. In this paper, the following method is devised: A removable alloy structural element is added to the top of the TDI system, consisting of six rectangular columns with different lengths, as shown in Figure 2. In addition, four calibration points are set up at the corners of the TDI light outlet. The coordinates $x_{b}$ of these 10 calibration points are known in the TDI body coordinate system. In the calibration experiment, the ground coordinates x of these 10 points are measured using total station. The ground coordinates and body coordinates of the 10 calibration points are substituted into the Equation (4), and the initial values of the datum transformation parameters $\left(X_{0},Y_{0},Z_{0},\omega ,\varphi ,\gamma \right)$ can be solved by least squares adjustment.

In the process of calibration, the datum transformation parameters determined by the benchmark points are treated as pseudo-observations with precise initial values, in which a priori precision was set up according to the nominal accuracy of total station. Meanwhile, $\theta_{x}$, $\theta_{y}$, and $\theta_{k}$ are also treated as pseudo-observations with zero initial values, in which a priori precision was set up by the nominal accuracy of corresponding scanning motor. All the error equations of pseudo-observations are combined with Equation (6) to construct the error equation system for TDI geometric calibration. The practical application shows that the adjustment iteration converges quickly, and the correlation between internal and external parameters is low.

It should be noted that during the calibration adjustment procedure, some of the extracted laser footprints within a planar target plate may be wrong or be omitted because of the limited accuracy of initial calibration parameters. To this end, in this paper, we design an algorithm that automatically extracts laser footprints within a planar target plate using its four corner points. In each adjustment iteration, laser footprints on the target plate are automatically extracted using the latest calibration parameters, ensuring that as much control information is utilized as possible.

## 4. Design and Construction of the Geometric Calibration Field Based on Planar Target Plates

In order to meet the needs of geometric calibration and accuracy verification of multiple sets of CE-3’s TDI equipment, a permanent outdoor calibration field was designed and built at Jiading Campus of Tongji University, and some high-precision plane target plates were used as control elements.

### 4.1. Requirements for the Spatial Orientation of the Target Plates

The spatial orientation of the plane target plate needs to be designed according to the characteristics of each system error source of TDI. For this purpose, the simulation data generated in the previous section are relied upon and each type of error is added separately in order to find the effect law of the ranging error $\Delta L_{k}$, scanning angle error $\Delta \theta_{x}$, and pointing error of $\Delta \theta_{y}$ on the laser footprint. The conclusions are as follows:

(1) The ranging error $\Delta L_{i}$ will distort the plane, the center area of the field of view has the greatest error on the $z_{b}$, and the edge of the field of view has a great influence on the coordinates of $x_{b}$ and $y_{b}$. Therefore, the correction of $\Delta L_{k}$ needs to set target plates in the center and edge of the field of view, and the target plates should have a certain depth in front and back.
(2) The scanning angle error $\Delta \theta_{x}$ will mainly cause the scene to pan in the $x_{b}$ direction (left and right directions), while it will cause a slope effect on the flat ground. Additionally, it is found in practice that $\Delta \theta_{x}$ is not the same for forward and reverse scanning, which causes a significant misalignment between adjacent strips. The placement of at least two left-right tilted target plates on the left and right sides of the field of view is required for controlling $\Delta \theta_{x}$.
(3) The pointing angle error $\Delta \theta_{y}$ mainly causes the scene to translate in the $y_{b}$ direction (up and down), and causes the ground to slope in the front and back directions. Therefore, at least two tilted target plates should be placed above and below the field of view. However, limited to the actual conditions of the experimental field, two tilted target plates can only be placed in the central field of view.

### 4.2. Target Board Layout Scheme

**Figure 3:**
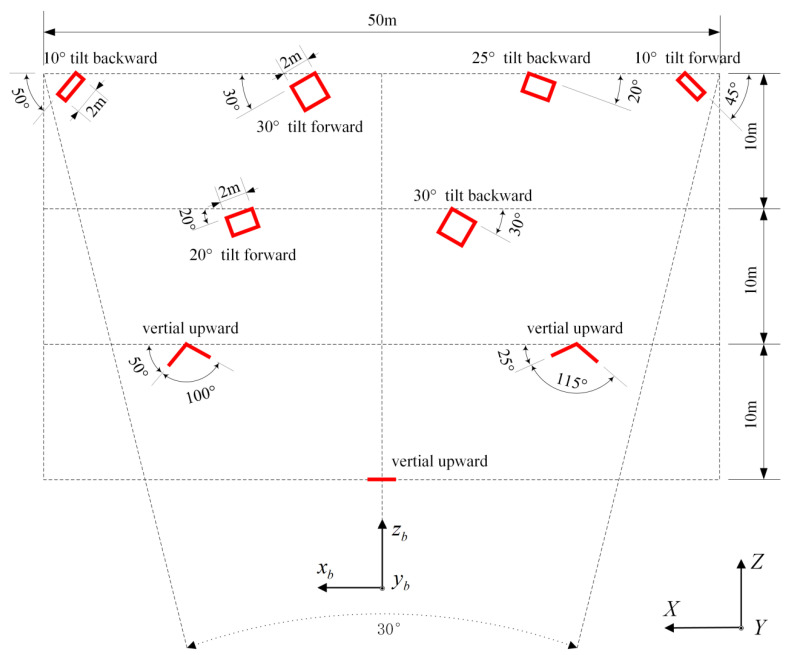
Layout scheme of flat target plates in the Jiading outdoor calibration field. This is a schematic diagram of the effect from the top view from the air. The red rectangle and line represent the flat target board, each is 2 meters wide and 4 meters high, $x_{b}$,$y_{b}$ and $z_{b}\;$ indicate the directions of the three coordinate axes of the TDI body coordinate system, *X*, *Y*, *Z* indicate those of the ground coordinate system. The *XZ* plane is basically parallel to the ground, and the *Y* axis is vertically upward. During geometric calibration, try to ensure that the orientation of the coordinate axes of the TDI body frame and the ground coordinate system is consistent. In order to effectively separate the influence of the TDI systematic error parameters, the azimuth and pitch angles of each flat target plate in the *XZ* plane are specially designed, and the corresponding angles are given in the figure.

According to the above analysis, in order to effectively separate various systematic errors, the layout scheme shown in Figure 3 was designed, which consistd of 11 flat target plates of 2 m wide and 4 m high. The orientation of each target plate was carefully designed and divided into four levels in the horizontal direction. The observatory where the TDI system is placed is about 100 m away from the furthest end, with a horizontal field of view of about 30°, close to the real working condition during lunar landing.

The parameters of these plane target boards are as follows: (1) size 2 m × 4 m, flatness better than 0.5 cm, metal material, and no mirror reflection; (2) each target board is evenly laid no less than 9 marker points (including the four corners of each target board) for total station measurement; (3) firm and not easily disturbed; (4) target boards and marker points have fixed numbers.

### 4.3. Coordinate System Design and Target Board Measurement

The ground coordinate system O-XYZ of the calibration field is designed as a right-handed coordinate system, and the three-axis orientation is as consistent as possible with the TDI body coordinate system $o-x_{b}y_{b}z_{b}$ to simplify the adjustment solution. The origin is fixed with cross steel nails and poured as a permanent marker. In this coordinate system, a Leica TS06 total station is used to measure all the reflective marker points on each flat target plate and on the TDI equipment itself with the accuracy of 2 mm.

## 5. TDI Intrinsic Parameter Calibration and Accuracy Assessment

### 5.1. Calibration Data Collection

In August 2012, the calibration and accuracy verification of TDI was carried out in the outdoor calibration field at the Jiading Campus. In order to cover the vertical field of view of TDI as much as possible, five scans of the calibration field were performed using TDI with pitch angles of −5°, 0°, and +5° (hereafter identified by the codes M5, Z0, and P5 respectively).

### 5.2. Intrinsic Parameter Calibration

Three groups of observation data at 0° elevation angle (Z0-3rd, Z0-4th, and Z0-5th) were randomly selected for error calibration, and others were used for accuracy verification. It is found in data preprocessing that the reflected signal intensity of the target plate closest to TDI is relatively large, and some channels are close to saturation resulting in unstable ranging data; therefore, this target plate is not used in later calibration calculation. In the end, there are 10 target plates actually used for system calibration and accuracy verification. 

In order to prove the necessity of carrying out the geometric calibration for CE-3’s TDI, Z0-3rd, Z0-4th, and Z0-5th, these three groups of observation data were used to generate point clouds in the TDI body coordinate system, and then the laser footprints corresponding to above 10 target plates were extracted and used to fit the plane equation. Finally, the distance between each laser footprint and the fitted plane was calculated, which reflects the coplanar error of laser footprints on each planar target plate. The maximum, minimum, and RMS error are summarized in Table 1.

It can be seen from the results in Table 1 that before the geometric calibration of the TDI intrinsic parameters is carried out, the error of the intrinsic parameters will produce a false obstacle with a maximum height of about 1.4 m on the plane, which will lead to the failure of the CE-3 obstacle avoidance system to find a suitable landing site or find a false flat area, and therefore, it will be difficult for the CE-3 soft landing mission to succeed. Therefore, it is necessary to carry out geometric calibration of the systematic errors of the TDI intrinsic parameters.

The calibration results of the constant error for scanning angle $\theta_{x}$ and direction angle $\theta_{y}$ are shown in Table 2, from which it can be seen that the constant errors of $\theta_{x}$ and $\theta_{y}$ obtained from these three groups of data are almost the same, and the deviation from each other is less than 10^−^^5^ rad, obviously, they are all systematic errors.

**Figure 4:**
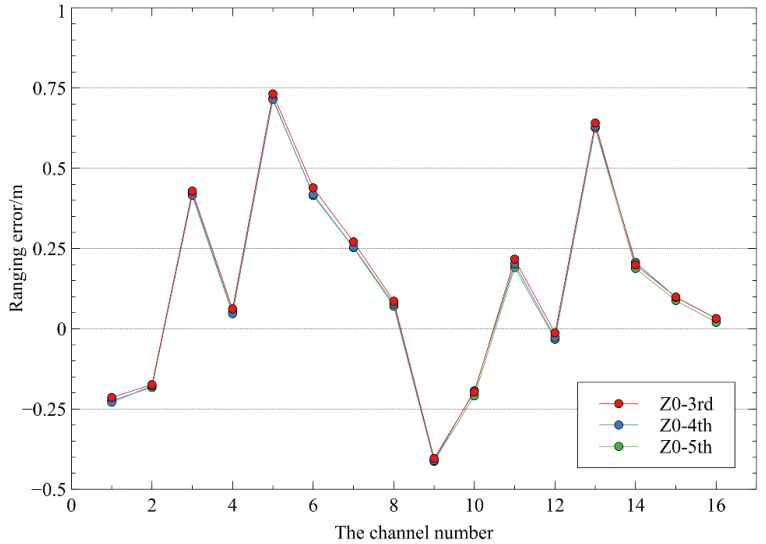
Calibration results of the 16-channel range error for CE-3’s TDI. The horizontal axis is the laser beam channel index number of TDI, and the vertical axis is the calibration result of the laser beam ranging error. Three sets of scanning data with zero degree elevation angle were used as input data for geometric calibration. Through comparison, it can be seen that the ranging errors obtained from three geometric calibrations are very consistent.

The calibration results of the range error for all the 16 channels of TDI are shown in Figure 4. From the figure, it can be found that the range errors of different channels vary greatly, and the maximum value exceeds 0.7 m, but also has good stability in multiple scans.

The above results also show that the systematic errors of CE-3’s TDI are relatively stable; therefore, it is feasible to improve the positioning accuracy by calibration. 

### 5.3. Validation of Calibration Results

Based on the laser point cloud of plane target boards, the absolute distance from the laser footprint to the target board and the relative plane fitting errors these two indicators are used to comprehensively verify the effectiveness of above calibration results.

#### 5.3.1. Absolute Accuracy Verification of the Same Elevation Angle

The absolute coordinates of the point cloud are used to verify the coplanar accuracy. Because the datum transformation from the TDI body frame to the ground coordinate system changes when TDI is at different pitch angles, the absolute accuracy verification can only be carried out in multiple sets of data collected at the same pitch angle.

The calibration results of Z0-3rd were selected, and the absolute coplanar error of Z0-4th and Z0-5th data were counted. The results are shown in Table 3. It can be seen that the coplanar RMS error is about 4 cm, which meets the accuracy requirement of CE-3’s TDI.

#### 5.3.2. Relative Accuracy Verification

Firstly, using the calibration results of Z0-3rd, three sets of original observation data were selected from M5 and P5 scanning data to generate point clouds in the body coordinate system, and then the point set corresponding to each target plate was extracted and used to fit the plane equation. Finally, the distance between each laser footprint and the plane was calculated and the relative coplanar error was calculated, as shown in Table 4. It can be seen that the coplanar accuracy of each target plate is better than 4 cm, which also meets the accuracy requirements of CE-3’s TDI.

#### 5.3.3. Cross-Validation of Different Time Phases

In order to verify the time validity of the August 2012 calibration results, the calibration field at the Jiading Campus was used again in March 2013 to verify the accuracy of the CE-3’s TDI, and five scans were taken at three elevation angles of −5°, 0, and +5°, in the same way.

The results of the Z0-3rd calibration in August 2012 were used again to calculate the relative coplanar error of the nine sets of data. The results are shown in Table 5, and it shows that the last calibration parameters are still valid. The coplanar error of each target plate for the nine sets of data is around 3 cm, indicating that the calibration results have good temporal validity.

## 6. Conclusions

In this paper, according to the design parameters and application scenarios of CE-3’s TDI, a simplified model of its direct positioning equation is constructed to achieve a balance between point cloud accuracy and on-board operation efficiency. By analyzing the error characteristics of parameters in TDI, a permanent outdoor calibration field based on planar target plate was designed and constructed. By establishing the virtual error equations of internal and external parameters, a robust calibration adjustment system is constructed. The calibration results of multi-measurement and multi-angle scanning data show that the intrinsic parameter error of TDI will produce a false obstacle with a maximum height of about 1.4 m on the plane, which will cause the obstacle avoidance system of CE-3 to fail to find a suitable landing area, or find a false flat area, and it will be difficult for the soft landing mission of CE-3 to succeed. Therefore, it is necessary to carry out geometric calibration of systematic errors in TDI intrinsic parameters. The intrinsic parameters of TDI have good stability and repeatability, and the accuracy of the reconstructed 3D surface can reach about 4 cm after systematic error correction, which can effectively support the on-board automatic identification of protruding obstacles, and provide reliable terrain support for the autonomous obstacle avoidance of CE-3 lander. The research in this paper provides a valuable obstacle avoidance scheme for soft landing of other sensors on extraterrestrial celestial bodies, and also provides a systematic solution for the geometric calibration of multi-channel liDAR.

## Figures and Tables

**Table 1:** Coplanar error statistics of laser footprints before geometric calibration.

	Number of Points	Min Error /m	Max Error /m	RMS Error /m
Z0-3rd	3055	0.000	1.401	0.401
Z0-4th	3492	0.000	1.427	0.380
Z0-5th	3137	0.000	1.356	0.399

**Table 2:** Calibration results of the constant error for the scanning angles of CE-3’s TDI.

	Z0-3rd	Z0-4th	Z0-5th
$\theta_{x}\;/\mathrm{mrad}$	−1.384	−1.362	−1.378
$\theta_{y\;}/\mathrm{mrad}$	−0.002	−0.002	−0.002

**Table 3:** Absolute coplanar accuracy after applying Z0-3rd calibration results.

Validation Data	Number of Target Plates	Number of Points	Coplanarity RMSE/cm
Z0-4th	10	3502	4.0
Z0-5th	10	3553	4.2

**Table 4:** Accuracy of coplanar fitting by applying the Z0-3rd calibration results.

Validation Data	Number of Target Plates	Number of Points	Coplanarity RMSE/cm
M5-1st	10	3210	3.2
M5-3rd	10	3180	3.2
M5-5th	10	3288	3.3
P5-1st	10	3426	3.0
P5-3rd	10	3523	3.1
P5-5th	10	3405	3.7

**Table 5:** Relative coplanar accuracy of cross-calibration at different measurement times.

Validation Data	Number of Target Plates	Number of Points	Maximum Deviation/cm	Coplanarity RMSE/cm
M5-1st	9	2957	14.5	3.3
M5-3rd	9	2949	11.2	3.1
M5-5th	9	2938	11.9	3.3
Z0-1st	9	2867	12.4	3.2
Z0-3rd	9	2916	11.5	3.0
Z0-5th	9	2969	11.5	3.1
P5-1st	9	3346	12.9	2.5
P5-3rd	9	3175	12.2	2.6
P5-5th	9	3157	12.7	2.6

## Data Availability

The data that support the findings of this study are available from the corresponding author upon request.
